# Regulation of Long Non-coding RNAs and MicroRNAs in Heart Disease: Insight Into Mechanisms and Therapeutic Approaches

**DOI:** 10.3389/fphys.2020.00798

**Published:** 2020-07-10

**Authors:** Lucy Collins, Pablo Binder, Hongshan Chen, Xin Wang

**Affiliations:** ^1^Division of Cardiovascular Sciences, Faculty of Biology, Medicine and Health, The University of Manchester, Manchester, United Kingdom; ^2^Key Laboratory of Cardiovascular and Cerebrovascular Medicine, Nanjing Medical University, Nanjing, China

**Keywords:** microRNAs, long non-coding RNAs, cardiovascular disease, therapy, proteostasis

## Abstract

Cardiovascular disease is the leading cause of mortality worldwide and there is an increasing need to identify new therapeutic targets that could be used to prevent or treat these diseases. Due to recent scientific advances, non-coding RNAs are widely accepted as important regulators of cellular processes, and the identification of an axis of interaction between long non-coding RNAs (lncRNAs) and micro RNAs (miRNAs) has provided another platform through which cardiovascular disease could be targeted therapeutically. Increasing evidence has detailed the importance of these non-coding RNAs, both individually and in an axis of regulation, in the processes and diseases involving the heart. However, further investigation into the consequences of targeting this mechanism, as well as refinement of how the system is targeted, are required before a treatment can be provided in clinic. This level of genomic regulation provides an exciting potential novel therapeutic strategy for the treatment of cardiovascular disease.

## Introduction

Cardiovascular disease is the leading cause of mortality worldwide. In 2016, it is estimated that cardiovascular disease caused 17.6 million deaths globally, an increase of 14.5% since 2006 (Benjamin et al., 2019). It is predicted that, without further medical innovation, by 2030 over 23.6 million people are expected to die from cardiovascular disease (AHA, 2019) the majority of which will be due to stroke and heart disease. Stress conditions cause alterations in cardiac homeostasis and result in pathological conditions such as cardiac hypertrophy, myocardial infarction, and heart failure. During the development and progression of these conditions, various cellular changes occur including cell death through both necrosis and apoptosis, extra cellular matrix deposition leading to cardiac fibrosis, and increased protein load that results in endoplasmic reticulum (ER) stress and proteotoxicity. Novel therapies are required to target these cellular events that occur during these conditions, both to prevent their occurrence and as treatment.

The majority (98%) of the genome consists of non-coding DNA, which was once considered ‘junk DNA.’ However, the finding that around three quarters of the genome has the potential to be transcribed (Djebali et al., 2012) demonstrated both the importance and potential of non-coding RNAs, and revolutionized how this part of the genome is viewed, bringing about new potential therapeutic strategies to treat disease. As will be discussed, numerous studies have identified regulatory roles of these non-coding RNAs in cardiovascular disease. More recently, the regulatory interplay between non-coding RNAs, specifically between long non-coding RNAs and micro-RNAs, has been identified, and this review will explore the potential therapeutic approaches which could target this regulatory system in diseases of the heart, focusing predominantly on those with evidence of their impact in animal models.

## Overview of lncRNAs and miRNAs

Non-coding RNAs were first identified in the 1960s though it took many more years to realize their potential in the cell (Cech and Steitz, 2014). They are functional RNAs that, unlike coding RNA, do not undergo translation, and it appears that their predominant role is gene expression regulation. MicroRNAs and long non-coding RNAs are the two most understood members of the non-coding RNA family and will be the focus of this review.

### MicroRNAs

MicroRNAs (miRNAs) are small, single-stranded, non-coding RNAs around 18–25 nucleotides in length which were first discovered in 1993 (Lee et al., 1993) in *Caenorhabditis elegans*. They regulate gene expression at the post-transcriptional level, either by directing their target mRNA to be degraded or inhibiting its translation. Over 2000 miRNAs have been identified in the human genome (Alles et al., 2019) and these are thought to regulate around one third of all genes.

Most miRNAs are transcribed by RNA polymerase II into primary miRNA (pri-miRNA) with a hairpin loop structure which often have a poly-A tail. Following this they undergo processing in the nucleus, which is enabled by interaction with DGCR8 and DROSHA, a ribonuclease, leading to the generation of a hairpin structure known as the pre-miRNA. This is subsequently translocated into the cytoplasm where it is further processed by DICER into miRNA duplexes. Duplexes unwind; one strand, known as the passenger strand, is degraded, whilst the other remains as the mature miRNA which is incorporated into the RNA-induced silencing complex (RISC), along with other proteins such as Ago-2, to form a multiprotein complex through which the miRNA interacts with mRNA.

MiRNAs function by binding to mRNA sequences with complementary base-pairs. The majority of miRNAs bind to the 3′ untranslated region of target mRNAs; however, interactions with other sites, such as promoters and 5′ regions have been described (O’Brien et al., 2018). Once bound, miRNAs cause silencing through multiple mechanisms including; (1) translational inhibition, often through interfering with ribosomal processes, (2) site-specific mRNA cleavage, commonly known as RNA interference (RNAi), and (3) destabilizing the mRNA through deadenylation leading to enhanced degradation. Whether mRNA is targeted for irreversible degradation or reversible translational inhibition is determined by the level of sequence complementarity. On top of their silencing capability, recent research in the past decade has identified that miRNAs can also function as ligands for toll-like receptors (TLRs), leading to their activation. This has been particularly investigated in cancer, where paracrine miRNA-TLR activation leads to cytokine production by local immune cells, promoting cancer proliferation and metastatic potential (Fabbri et al., 2013). Furthermore, miRNAs have been shown to regulate gene expression independent of RISC, demonstrating the complexity in their functions (Catalanotto et al., 2016).

MiRNAs are highly evolutionarily conserved and play key roles in various biological processes including cell survival, proliferation, angiogenesis, and insulin secretion, as well as many others, and as such, are key regulators of, and thus therapeutic approaches for, diseases such as cancer and cardiovascular disease (Di Leva and Croce, 2015; Oliveira et al., 2016; da Silva et al., 2018).

### Long Non-coding RNAs

Long non-coding RNAs (LncRNAs) are characterized as RNAs longer than 200 nucleotides. Unlike miRNAs, lncRNAs often have poor sequence conservation and expression, and are more numerous. The first eukaryotic lncRNA was identified in the 1990s - H19 (Brannan et al., 1990). Potentially a reason for its early discovery is that H19, unlike many other lncRNAs, has high sequence conservation among mammals. However, H19’s functional ability was not determined at this time, and the first lncRNA to be identified with a specific function was Xist, which is involved in X chromosome inactivation (Brockdorff et al., 1991; Brown et al., 1991). The publishing of the human genome in 2001 led to a greater focus on non-coding RNAs, which in turn led to a substantial increase in the number of lncRNAs identified (Jarroux et al., 2017).

LncRNA synthesis, much like that of mRNA, occurs by RNA polymerase II dependent transcription and can then be further processed, for instance by splicing and polyadenylation. Their structure, too, is similar to that of mRNA, so much so that they were originally known as ‘mRNA-like’ (Erdmann et al., 1999), though they lack a stable open reading frame. As a whole, they have no specific localization, and can be localized in the nucleus and/or the cytosol. LncRNAs are a heterogeneous group, grouped into classes with varying functions, which are explored further in other reviews (Jarroux et al., 2017). The majority of lncRNAs appear to function as gene regulators, influencing both peri- and post-transcriptional gene expression.

LncRNAs can directly bind or interact with mRNA leading to translational suppression (Yoon et al., 2012) or mRNA decay (Gong and Maquat, 2011). LncRNAs can also bind proteins, altering their localization, activity, stability, and can also sequester them (Paneru et al., 2018). There are five main mechanisms through which lncRNAs have been identified to function and alter gene expression, and individual lncRNAs are not restricted to one mode of action.

#### Scaffolds

Scaffold lncRNAs provide a platform at a specific genomic locus. They contain multiple binding sites, allowing the recruitment of numerous molecules that can form a functional complex, known as a ribonucleoprotein (RNP) complex. TUG1 can bind to the methylated polycomb 2 protein, leading to assembly of a transcription repressor complex, which is believed to be important for grown-control gene repression (Yang et al., 2011).

#### Decoy

LncRNAs can also act as decoys for transcription factors or other regulatory proteins, competing for binding sites, preventing them from acting on the loci of interest. The lncRNA p21-associated ncRNA DNA damage activated (PANDA) is a well-studied lncRNA acting as a decoy following DNA damage. PANDA binds to the transcription factor NF-YA, which causes its sequestration, preventing it from binding and promoting the transcription of pro-apoptotic genes (Hung et al., 2011).

#### Signals

Signal lncRNAs are transcribed following a specific stimulus, acting in a time- and space-dependent signaling pathway. This mechanism is evident in phenomena such as gene imprinting (Pandey et al., 2008; Mohammad et al., 2009). It appears that the benefit of this mechanism is that, as it is RNA-dependent, it allows for a faster response due to the lack of need for protein translation.

#### Guides

LncRNAs that act as guides are required to direct transcription-related factors to the correct genomic loci. They can act in either a *cis* or *trans* fashion. For instance, the lncRNA HOTAIR directs PRC2, a chromatin modifier with histone methyltransferase activity, to the HOXD locus, leading to gene silencing of these transcription factors. HOTAIR has been identified as an oncogene, promoting cancer metastasis through this guide capability (Gupta et al., 2010). As well as acting as guides to supress transcription, lncRNAs can also promote transcription by guiding transcription factors and histone modifiers to gene targets. For instance, the lncRNA HOTTIP has been identified as recruiting the MLL protein to the 5′HOXA locus through binding to the WDR5 adaptor protein, leading to H3 lysin 4 trimethylation, and subsequent gene transcription (Wang et al., 2011).

#### Enhancers

A less understood mechanism of lncRNAs in altering gene expression is by acting as enhancers. These lncRNAs have been shown in human cell lines to Ørom et al. (2010) to regulate gene expression distant to the lncRNA location. More research is required into the exact mechanism, though one study suggests that this occurs through DNA looping to bring the enhancer and the target site into closer proximity, and promoting the recruitment of RNA polymerase II and transcription factors leading to target gene expression (Lai et al., 2013).

## The lncRNA–miRNA–mRNA Axis

**FIGURE 1:**
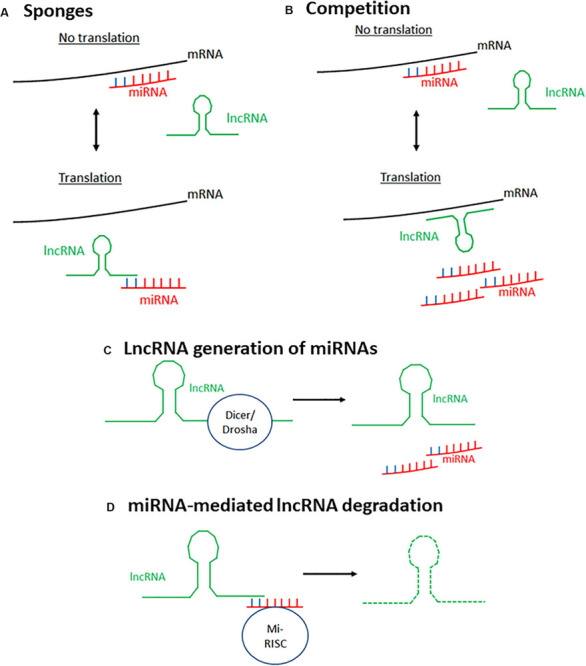
Mechanisms of lncRNA–miRNA interaction to affect mRNA expression. There are four main known mechanisms through which lncRNAs and miRNAs interact to alter gene expression. **(A)** LncRNAs can act as sponges for miRNAs. LncRNAs bind miRNAs preventing their interaction with their target mRNA, thus preventing silencing of this gene. **(B)** LncRNAs and miRNAs compete to bind the same target mRNA, thus, depending on the stimulus, gene expression can be differentially expressed. **(C)** miRNAs can be generated from lncRNAs through spicing of the lncRNA by endoribonucleases such as Dicer and Drosha. **(D)** MiRNAs silence lncRNA activity by targeting lncRNAs for degradation using the same mechanism through which they target mRNAs. Mi-RISC, miRNA-induced silencing complex.

Whilst many lncRNAs and miRNAs have been identified separately as being involved in biological functions and disease, they have also been shown to be involved through interacting with one another, resulting in another level of gene regulation. This has been termed the lncRNA–miRNA–mRNA axis. This regulation is bi-directional, with miRNAs influencing lncRNA expression, and vice versa. Multiple mechanisms of lncRNA–miRNA interaction have been reported (Yoon et al., 2014) (Figure 1) and are discussed below.

### LncRNAs Acting as Sponges

LncRNAs can bind miRNAs, preventing them from interacting with their target mRNA, therefore acting as positive regulators of transcription. Those that do this are known as competitive endogenous RNAs (ceRNAs), decoys, or sponges as this review will call them. Most sponge lncRNAs bind to miRNAs through complementary interaction between the Ago binding sites on miRNAs and the 3′ ends of lncRNAs. Cardiac hypertrophy related factor (CHRF) was the first lncRNA described as able to act as a sponge, directly binding to miR489 to downregulate its expression, with enforced CHRF overexpression leading to reduced miR-489 expression and a hypertrophic response in neonatal cardiomyocytes regardless of the presence of a pro-hypertrophy stimulus (Wang et al., 2014a). Since this discovery, other lncRNAs have been demonstrated to have this ability, such as SNHG7 which was found to sponge miR-216b in order to promote proliferation and metastasis of cancerous colorectal cells (Shan et al., 2018). However, on top of directly binding to miRNAs to prevent their interaction with mRNA, lncRNAs have also been identified as able to bind mRNAs to prevent miRNA-mediated degradation of both the mRNA and lncRNA (Barbagallo et al., 2018).

### Competition Between miRNAs and lncRNAs

It has been noted that miRNAs and lncRNAs can compete to bind the same target mRNA. Faghihi et al. (2010) identified this phenomenon *in vitro* in the regulation of the *BACE1* gene. They demonstrated that miR-485-5p and the lncRNA BACE1AS, which is partially antisense to *BACE1* mRNA, compete to bind the same mRNA region of BACE1, with BACE1AS overexpression protecting BACE1 from mRNA-mediated silencing. This suggests that the cellular stimulus and the ratio between miRNAs and lncRNAs may determine whether a gene is expressed or silenced.

### MiRNA Generation by lncRNAs

As well as acting antagonistically, lncRNAs have been shown as able to be processed to produce miRNAs. The first exon of lncRNA H19 contains the transcript for miR-675, and H19 has been shown to effectively act as a pri-mRNA for miR-675. Processing of H19 to release this miRNA is regulated by the RNA binding protein HuR, upregulated during stress, with HuR inhibiting miR-675’s processing by binding to full-length H19 (Keniry et al., 2012). Removal of HuR allowed the processing of H19, and it is believed to be due to the removal of HuR’s inhibition of the endonuclease Drosha. This allows for temporal- and spatial-specific regulation, with this miRNA released prior to birth to prevent placental overgrowth. It has also been shown to be important in skeletal muscle differentiation and regeneration (Dey et al., 2014). Further to this, the lncRNA Ang-362 and miR-221/222 were shown to be co-transcribed in vascular smooth muscle cells (Leung et al., 2013) with these microRNAs transcribed as part of the larger transcript of Ang-362 that lies proximal. Using siRNA-mediated knockdown of the lncRNA, this group confirmed that expression of Ang-362 is required for expression of these miRNAs, and this is important for Angiotensin II-mediated cellular proliferation. The finding that lncRNAs can positively regulate miRNAs led to the re-analysis of our understanding of the relationship between non-coding RNAs.

### MiRNA-Triggered lncRNA Decay

As previously described, miRNAs can initiate mRNA decay by recruiting the RNA-induced silencing complex (RISC). Similarly, miRNAs can trigger lncRNA decay. As with mRNA decay, the miRNA binds the lncRNA as part of the RISC due to imperfect base-pair complementarity. This interaction targets the lincRNA for degradation. MALAT1, one of the most abundant and conserved lncRNAs, is degraded through direct binding with miR9 in the nucleus (Leucci et al., 2013). Knockdown of Ago2 led to elevated levels of MALAT1, indicating that this degradation is through interaction with RISC. The let-7 family of miRNAs has been shown to promote the degradation of multiple lncRNAs, including HOTAIR (Yoon et al., 2013) and lincRNA-p21 (Yoon et al., 2012) through the RBP HuR.

### Novel Mechanisms of Interaction

As well as the more developed concepts of lncRNA–miRNA–mRNA interaction described above, other ways in which lncRNAs and miRNA have been shown to interact have been identified. Yu et al. (2017) identified that the lncRNA CCAT2 could regulate miR-145 expression by selectively blocking its maturation. This demonstrates that there are potentially other mechanisms of RNA-mediated regulation, suggesting that this axis may be more complex than first thought.

## Role of Long Non-Coding RNAs and Micro RNAs in Heart Disease

Emerging evidence has highlighted the important roles of miRNAs and lncRNAs in heart development, physiology and disease. Transcriptome and functional analyses have identified numerous miRNAs and lncRNAs whose expressions are altered in heart pathology, and revealed that while the expression of some of these non-coding RNAs show negative effects in the progression of disease, others appear to play protective roles (Thum et al., 2008; Di Mauro et al., 2018). Therefore, greater understanding is required on the relationship between non-coding RNAs and the pathogenesis and development of heart disease. This, together with discovery of novel approaches to manipulate their expression and activity, will aid the design and development of novel diagnostic and therapeutic strategies to ameliorate cardiac dysfunction and diminish the pathological progression of heart failure.

The role of miRNAs and lncRNAs in cardiovascular disease has previously been described in detail (Uchida and Dimmeler, 2015; Wronska et al., 2015) and both lncRNAs and miRNAs have been shown to act as both positive and negative regulators of cardiovascular disease (Sardu et al., 2014).

### Role of Non-coding RNAs in Cardiac Hypertrophy

Cardiac hypertrophy is the adaptive response to stress-induced injury due to pathological pressure overload or neurohormonal stimulation (Frey and Olson, 2003). This response is initially compensatory and aims to reduce wall stress through thickening of the ventricular wall in order to maintain normal cardiac function. However, sustained hypertrophy often leads to maladaptive cardiac remodeling which can progress into heart failure.

During heart hypertrophy and dysfunction diverse signaling pathways are activated resulting in increased cardiomyocyte size, protein synthesis, organelle alterations, and expression of fetal genes (Nakamura and Sadoshima, 2018). However, the molecular mechanisms responsible for the development of cardiac hypertrophy are still not fully understood. Several studies have shown miRNAs and lncRNAs to be key players in the regulation of cell differentiation, growth, survival and proliferation. Particularly, many of these non-coding RNAs have been reported to be critical regulators in heart hypertrophy and failure in *in vivo* and *in vitro* disease models (Kumarswamy and Thum, 2013).

The involvement of miRNAs in heart hypertrophy was firstly described in cardiac tissue from mice in response to pressure overload and activated calcineurin overexpression, which result in pathological cardiac remodeling. Initially, more than a dozen miRNAs showing up- or down-regulation were identified, many of which were similarly altered in failing human hearts (van Rooij et al., 2006). One of these, miRNA-195, is up-regulated during cardiac hypertrophy and its cardiac overexpression appears to be sufficient to drive cardiac pathological hypertrophy leading to heart failure in mice. This revealed a crucial role for miRNAs in hypertrophic growth leading to remodeling of the heart as a result of pathological signaling. The same group reported that the cardiac-specific miRNA, miR-208, conserved among humans, mice, rats, and dogs is required for hypertrophy, fibrosis, and βMHC upregulation in cardiomyocytes under stress conditions (van Rooij et al., 2007, 2008).

On the other hand, miR-133 and miR-1 are downregulated in mouse and human models of cardiac hypertrophy. Overexpression of both miRNAs inhibits cardiac hypertrophy, and inhibition of miR-133 causes significant cardiac hypertrophy and cardiac dysfunction via upregulation of the miR-133 targets Rho1, Cdc42, and Nelf-A/WHSC2 *in vivo*, suggesting miR-133 is cardioprotective in the context of hypertrophy (Carè et al., 2007). Several other miRNAs have been described as modulators of cardiac hypertrophy, either by protecting cardiac tissue from pathological hypertrophy such as miR-1 and miR-541 (Liu et al., 2014) or by contributing to hypertrophy, such as miR-23a and miR-34 (Li et al., 2015a). This further highlights the importance of the regulation of miRNAs as potential targets for therapeutic application in heart disease.

LncRNAs have also been shown to have a role in the development of cardiac hypertrophy. Viereck et al. (2016) identified the lncRNA cardiac hypertrophy-associated transcript (Chast) and showed it to be increased in mice in response to pressure overload. This group showed, both *in vitro* and *in vivo*, that Chast can induce cardiac hypertrophy. Furthermore, the human homolog CHAST is upregulated in human samples of hypertrophic cardiac tissue, thus suggesting a conserved role. Interestingly, as well as lncRNAs being able to interact with chromatin remodeling factors and alter chromatin structure, chromatin remodeling factors have been shown to alter lncRNA expression, demonstrating a feedback loop that creates another level of regulation, and one such feedback loop, involving the lncRNA Mhrt and chromatin has been shown to be important for heart function and protecting the heart from pathological cardiac hypertrophy (Han et al., 2014). Mhrt is a cardiac-specific lncRNA that antagonizes the chromatin-remodeling factor Brg1. During hypertrophy, stress causes Brg1 activation, stimulating the Brg1-Hdac-Parp chromatin repressor complex, which results in reduced cardiac Mhrt expression. Restoration of Mhrt expression protects the heart from cardiac hypertrophy and heart failure.

### Role of Non-coding RNAs in Arrhythmias

As well as influencing structural cardiac changes, lncRNAs and miRNAs have also been implicated in the development of arrhythmias (Kim, 2013; Zhang Y. et al., 2019). MiRNAs in particular have been implicated in the development of atrial fibrillation (AF) (Santulli et al., 2014) the most common sustained cardiac arrhythmia seen in clinic (Lee et al., 2014). Overexpression of miR-328, which is upregulated in tissue samples from AF patients, leads to AF in mice due to a reduced L-type Ca^2+^ current (Lu et al., 2010). AntagomiR-mediated reduction in miR-328 levels reverses the AF phenotype, demonstrating its importance in regulating calcium signaling and atrial electrical conduction during AF. Alternatively, some miRNAs become downregulated during certain cardiac arrhythmias. Downregulation of mir-1 during disease results in tachyarrhythmia due to loss of its ability to regulate Connexin 43 (Cx43), leading to Cx43 hyperphosphorylation and displacement (Curcio et al., 2013). This demonstrates the varied roles that miRNAs play in regulating the electrical remodeling that occurs in the heart during disease, and the varied ways in which they could be manipulated for therapeutic purposes.

However, the ability of miRNAs to alter cellular processes leading to arrhythmias has presented difficulties when targeting miRNAs for treatment. In one study, overexpression of human miRNA-199a in pig hearts following myocardial infarction initially seemed to be a therapeutic success, with treated animals showing improved contractility and reduced infarct size due to the ability of this miRNA to promote cardiomyocyte proliferation (Gabisonia et al., 2019). However, the continued expression of miR-199a resulted in sudden arrhythmic death in the majority of animals. Evidently, this must be taken into account and doses carefully monitored should these molecules be targeted for therapeutic benefit, in order to reduce adverse effects.

### Role of Exosomal miRNAs in Cardiovascular Disease

As well as the canonical pathway for miRNA gene regulation, miRNAs can also be secreted and transported throughout the body in extracellular vesicles known as exosomes; these are known as exosomal miRNAs Valadi et al., 2007). Encapsulation in exosomes protect these miRNAs from degradation by RNases (Mitchell et al., 2008; Koga et al., 2011). These miRNAs are important in cell–cell communications and regulate gene expression in recipient cells. Exosomal miRNAs have been implicated in the development of diseases such as cancer (Melo et al., 2014) and diabetes (Santulli, 2018), and viruses can promote their release in order to dampen the immune response (Pegtel et al., 2010). Similarly, exosomal miRNAs have been shown to prevent against cardiac injury induced during sepsis (Wang et al., 2015c). The exact mechanism by which miRNAs are sorted and transported into exosomes within a cell is unclear; however, it appears that this is a regulated process and not random, considering that miRNAs in exosomes differ to the general miRNA content in the parent cell (Goldie et al., 2014). Exosomal miRNAs have been implicated in CVDs (Chistiakov et al., 2016) as will be discussed below, but the complexity in how they are regulated, and the low exosomal yield, means that much more research is required to allow for effective therapeutic strategies involving them (Gangadaran et al., 2018).

Exosomal miRNAs have been shown to regulate development of fibrosis following myocardial infarction. Morelli et al. (2019) identified that exposure of fibroblasts to cardiomyocyte-derived exosomes isolated from a murine model of myocardial infarction were sufficient to initiate myofibroblast differentiation and activation, whilst exosomes derived from sham mice had no effect. They identified that exosomal transport of miR-195 was significantly upregulated, and inhibition of miR-195 using a miRNA mimic prevented the upregulation of periostin, a myofibroblast marker, in cultured fibroblasts. Other exosomal miRNAs have also shown to influence fibrotic development following MI. Exosomes isolated from mice subjected to ligation of the left anterior descending coronary artery (LAD) contained the miR-92a, which was also found to be upregulated in isolated cardiac myofibroblasts (Wang et al., 2020). These exosomes were shown to be cardiomyocyte-derived and, when introduced to isolated fibroblasts led to suppression of SMAD-7, a known inhibitor of alpha-smooth muscle actin (αSMA) which is crucial for myofibroblast differentiation and activation post-MI. The importance of exosomal miRNA secretion was confirmed *in vitro* using an inhibitor of exosome secretion. However, cardiac exosomes have also been shown to be enriched for miRNAs that have anti-fibrotic roles, such as miR-29a (Yamaguchi et al., 2015) and miR-144 (Li et al., 2014) and have shown to be cardioprotective during remote ischemic conditioning. These findings demonstrate the importance of investigating exosomal miRNAs as potential therapeutic targets for targeting cardiac fibrosis. Considering that this structural change in the heart is found in many cardiac pathologies, including myocardial infarction and cardiac hypertrophy, and since its occurrence is strongly related to progression to heart failure, it could provide an important therapeutic approach to prevent the development of this disease.

Other studies have demonstrated the involvement of exosomal miRNAs in other cellular responses during CVD. Pro-angiogenic miRNAs have been identified in exosomes from patient-derived cardiac progenitor cells (Barile et al., 2014), and rats injected with these exosomes had improved cardiac function, reduced cardiomyocyte apoptosis and increased angiogenesis after MI compared to controls. However, as with other cellular processes and with cell-resident miRNAs, miRNAs can have opposing effects, and some have been shown to promote cell death in the heart. The serum of acute myocardial infarction (AMI) patients had elevated levels of miR-30a, and *in vitro* investigation showed that upon exposure to hypoxic conditions this miRNA is upregulated and packaged into exosomes (Yang et al., 2016). Inhibition of exosome release, or miR-30a itself, led to autophagy of cultured cardiomyoblasts but attenuated apoptosis, demonstrating that this miRNA is important in regulating the cell survival response in cardiomyocytes following AMI. Considering that following AMI up to 1 billion cardiomyocytes are thought to undergo cell death (Murry et al., 2006) further investigation into the mechanism of this exosomal miRNA and how it might be targeted therapeutically could prove beneficial in reducing cardiac damage following AMI.

Exosomal miRNAs also influence CVDs of the vasculature and targeting their release could prove beneficial in treating pulmonary arterial hypertension (PAH). Sindi et al. (2020) identified that during the pre-clinical phase of PAH downregulation in the release of exosomal miRNAs miR-181a-5p and miR-324-5p occurs. These exosomal miRNAs are secreted in a paracrine fashion from endothelial cells and act to suppress vascular remodeling associated with PAH, such as increased proliferation and angiogenesis. In PAH mice, addition of these miRNAs assuaged disease progression. Crucially, this study identified that exosomal release of the miRNAs is mediated by the transcription factor KLF2, and as such provides a further information that could be used to target this pathway therapeutically.

The ability of exosomal transport of RNAs demonstrates the potential for miRNA–lncRNA axis interaction in cell types in which one component of the axis is not normally expressed. This presents a potential increased level of complexity in the regulation of gene expression across various cell types, and must be taken into account when investigating the possibility to exploit this axis for therapeutic benefit.

### Role of Non-coding RNAs as Biomarkers

Due to their involvement in cardiovascular disease, and their stability in plasma because of their ability to be confined in exosomes, greater interest is being placed on using miRNAs as biomarkers for disease (Zhou et al., 2018) and could also be used to influence treatment decisions (Kiyosawa et al., 2020). For instance, circulating levels of miRNAs -27a, -16b, -30e, 18b, and others are reduced in patients with heart failure, and some have been associated with an increased risk of mortality (Colpaert and Calore, 2019). This is not a unique phenomenon and, similarly, changes in circulating miRNAs have been shown to occur in other cardiovascular diseases such as acute myocardial infarction (Chistiakov et al., 2016) and cardiogenic shock (Jäntti et al., 2019). The increased use of bioinformatic modeling has aided the identification of potential miRNAs that may prove to act as biomarkers for multiple cardiovascular diseases (Kayvanpour et al., 2020).

A large body of research has focused on the investigation of miRNAs as potential biomarkers for myocardial infarction and related events. In patients with ST-segment elevation myocardial infarction (STEMI) following a type 1 MI, miR-331 and miR-151-3p were identified as significantly upregulated in these patients, and it appeared that their upregulation occurred as early as plaque rupture, suggesting that these could be used to predict STEMI development soon after the event, allowing for early intervention (Horváth et al., 2020). miRNAs have also been identified as predictive markers for cellular changes and remodeling following MI and reperfusion therapies such as percutaneous coronary intervention (PCI) and coronary artery bypass grafting (CABG). For instance, an increase in circulating levels of miR-320a has been noted to be correlated with development of adverse remodeling, such as wall thinning and dilation of the LV after PCI, which can promote the progression to heart failure (Galeano-Otero et al., 2020). Khan et al. (2020) have carried out a thorough analytical review of research to date surrounding the levels of miRNAs in blood or tissue extracted during CABP to predict the development of atrial fibrillation after CABG. Whilst many small studies have found increased levels of certain miRNAs correlated to arrhythmia development, the authors recognize that these have not been validated elsewhere in the literature, and many do not take into account changes in miRNA levels that may occur upon reperfusion. Evidently, further research is required to validate these miRNAs as biomarkers.

LncRNAs have also been investigated for their potential use as biomarkers of cardiovascular disease (Chen C. et al., 2019) and levels of lncRNAs have been shown to be identifiable in urine and blood plasma samples (Zhou et al., 2015; Terracciano et al., 2017) as they too can be encapsulated in exosomes (Li et al., 2015b). The lncRNAs NRON and MHRT have been identified as potential predictive biomarkers of heart failure (Xuan et al., 2017) and lncRNAs have also been identified as novel biomarkers for atrial fibrillation (Xu et al., 2016). Plasma levels of the lncRNA long intergenic non-coding RNA predicting cardiac remodeling (LIPCAR) were shown to predict survival in patients with heart failure, and higher levels of LIPCAR were associated with increased levels of left ventricular remodeling (Kumarswamy et al., 2014). LIPCAR also been associated with increased risk of coronary artery disease alongside the lncRNA H19 (Zhang et al., 2017). However, like studies into miRNAs, due to the novel exploration into this many studies have been conducted on small cohorts and thus need to be validated in larger cohort studies.

### Role of Non-coding RNAs in Cardiac Proteotoxicity

Due to the poor replicative ability of cardiomyocytes, it is highly important that cellular homeostasis is maintained, and as such there is a reliance on the protein quality control system. In recent years, alterations in proteostasis have received substantial attention and there is growing evidence for its role in the pathogenesis and progression of many forms of cardiovascular disease (Henning and Brundel, 2017). During cardiac remodeling, there is sustained alteration in protein homeostasis as a result of enhanced protein translation, oxidative stress, hypoxia and altered glucose metabolism, and this can result in the accumulation of misfolded protein, proteotoxicity and ER stress, and ultimately impacts cardiomyocyte function (Schirone et al., 2017). To cope with unfolded and misfolded proteins, activation of the unfolded protein response (UPR) takes place. The UPR acts as a defensive mechanism, that can protect cardiac cells functions by maintaining ER and protein homoeostasis by restoring its protein folding and clearance capacity and promoting cell survival. However, if the UPR fails to restore homeostasis, prolonged ER stress will eventually result in cardiomyocyte dysfunction and apoptosis, ultimately leading to cardiac disease (Kim et al., 2008).

There is substantial evidence of alterations in protein homeostasis resulting in ER stress in hearts exposed to pressure-overload and ischemic injury, and several studies in both mice and humans have provided evidence to suggest that the UPR response is essential in the development of these cardiac conditions. For example, ATF6 activation found to occur in cardiac hypertrophy is a protective cardiomyocyte response (Toko et al., 2010). Conversely, in mouse models of heart hypertrophy by pressure overload, elevated CHOP (Fu et al., 2010) and ASK1 (Liu et al., 2009) the downstream apoptotic effectors of the PERK and IRE1 branches of the UPR, respectively, have been reported. Similarly, under sustained ischemia or hypoxia, UPR related proteins ATF6, Bip, XBP1, ATF4, eIF2α, Tribbles 3, PUMA and CHOP are upregulated at both the mRNA and protein levels in cardiac cells, and in failing human hearts activation of the IRE1 and Perk branches can be observed (Wang et al., 2018a). Increased ER stress and alterations in the UPR have been reported in many cardiomyopathies including diabetic cardiomyopathy, alcoholic cardiomyopathy, cancer chemotherapy-induced cardiotoxicity, obesity-induced cardiomyopathy and autoimmune cardiomyopathy. Ultimately, chronic UPR activation contributes to the progression from compensatory hypertrophy to dilated cardiomyopathy and ER stress- dependent cell death participates in cardiac tissue decline in the progression of heart failure (Minamino et al., 2010).

Evidently, understanding the regulation of the signaling pathways involved in the UPR and the discovery of novel approaches to alleviate ER stress represents a promising strategy to prevent and treat cardiac dysfunction. Genetic and pharmacological regulation of these pathways have been shown be promising alternatives in the treatment of cardiac hypertrophy, MI and heart failure (Wang et al., 2018a). However, despite the great progress in understanding the role of ER stress in heart disease, the molecular mechanisms involved in the regulation of the pro-survival UPR pathway are still poorly understood. The recent discovery of non-coding RNAs as key players in the regulation of ER function in the heart suggests that further knowledge of regulatory mechanisms of non-coding RNAs in protein homeostasis could provide not only novel insight into the pathogenesis of cardiac disease under stress conditions but also lead to new therapeutic approaches.

It has been postulated that non-coding RNAs might be responsible for the regulation of the pro-survival/pro-apoptotic molecular switch observed in the UPR under sustained ER stress (Byrd and Brewer, 2013). Thus, modulation of miRNAs and lncRNAs particularly involved in the modulation of ER stress responses has the potential to be targeted to treat cardiovascular diseases. For instance, forced expression of miR-93 results in a decrease in ER stress-associated cell death and protects cardiomyocytes during I/R injury through suppression of PTEN and enhancement of Akt activity (Ke et al., 2016). In contrast, in an MI model, MiR-711 appears to increase ER-dependent apoptosis via downregulation of calnexin. Increased miR-711 results in an enhanced UPR response evidenced by upregulation of GRP78, ATF6, and spliced XBP1, and sustained expression of miR-711 eventually results in the increase of ER-stress-regulated apoptotic effectors, highlighting the substantial effect that miR-711 has as a ER stress-mediated apoptosis regulator in cardiac remodeling after MI (Zhao et al., 2018). Additionally, it has been shown that, *in vitro*, I/R triggers the expression of the lncRNA UCA1 which is accompanied by increased ROS production and ER stress, which leads to increased cardiomyocyte apoptosis (Chen J. et al., 2019). Overexpression of UCA1 seems to have a protective effect in cells subjected to I/R by modulating the ER stress response, resulting in reduced mitochondria dysfunction and apoptosis. Overall, this evidence suggests that modulation of non-coding RNAs have the potential to serve as therapeutic approach for the regulation of ER stress responses in the heart.

The impact of the dysregulation of non-coding RNAs on cardiac hypertrophy and MI, I/R and heart failure confirms that any imbalance or alterations in the expression and activity can have detrimental effects on cardiac function. It also highlights the potential non-coding RNAs present as future therapeutic targets in cardiovascular disease.

## Role of the lncRNA–miRNA Axis in Cardiovascular Disease

Increasing evidence has demonstrated that the functional interaction between miRNAs and lncRNAs has a critical role in cardiovascular disease (Table 1), some of which have been described elsewhere (Guduric-Fuchs et al., 2012; Li et al., 2019). As described above, there are several ways in which these types of non-coding RNAs can regulate each other’s activities and multiple mechanisms of lncRNA–miRNA interaction have been reported (Yoon et al., 2014). Unfortunately, information from research in cellular and animal models are so far limited and only a small number of lncRNA–miRNA–mRNA axis regulation and their molecular mechanism have been well studied. Here, we discuss some examples on miRNA–lncRNA interactions, focusing on their role in cardiac hypertrophy and ischaemic heart disease.

**TABLE 1:** Examples of miRNA–lncRNA–mRNA axis interactions in Cardiac disease.

	**Experimental disease model**	**lncRNA, response**	**miRNA**	**Interaction**	**Target**	**Function**	**References**
**Hypertrophic heart disease**	TAC angiotensin II	CHAR downregulated	miR-20b	Competitive RNA	PTEN	Anti-hypertrophic response in the heart	Zhang M. et al., 2019
	Angiotensin II heart failure	CHRF upregulated	miR-489	miRNA sponge	Myd88	Pro-hypertrophic response in the heart	Wang et al., 2014a
	ISO injection	CHRF upregulated	miR-93	Competitive RNA	AKT3	Pro-hypertrophic response in the heart	Wo et al., 2018
	TAC	CYTOR upregulated	miR-155	miRNA sponge	IKBKE	Anti-hypertrophic response in the heart	Yuan et al., 2019
	TAC ISO	H19 upregulated	miR-675	miRNA coding lncRNA	CaMKIIδ	Anti-hypertrophic response in the heart	Liu et al., 2016
	Angiotensin II	MIAT upregulated	miR-93	miRNA sponge	TLR4	Pro-hypertrophic response in the heart	Zhu et al., 2016
	ISO	MIAT upregulated	miR-150p	Not specified	P3000	Pro-hypertrophic response in the heart	Zhu et al., 2016
	TAC angiotensin II	Plscr4 upregulated	miR-214	miRNA sponge	Mfn2	Anti-hypertrophic response in the heart	Lv et al., 2018
	TAC PE	ROR upregulated	miR-133	miRNA sponge	ANP/BNP*	Pro-hypertrophic response in the heart	Jiang et al., 2016
**Ischemic heart disease**	MI	CARL upregulated	miR-539	miRNA sponge	PHB2	Enhanced Cardiac apoptosis and mitochondrial dynamics	Wang et al., 2014b
		H19, downregulated	miR-103/107	miRNA sponge	FADD	Inhibition of myocardial necrosis	Wang et al., 2015a
		MALAT1, upregulated	miR-145	miRNA sponge	Furin	Pro-fibrotic response in the heart	Huang et al., 2019
		MIAT, upregulated	miR-24	miRNA sponge	Furin	Pro-fibrotic response in the heart	Qu et al., 2017
		n379519, upregulated	miR-30	miRNA sponge	Collagen I/III*	Pro-fibrotic response in the heart	Wang et al., 2018c
		PFL, upregulated	Let-7d	competitive endogenous RNA	Ptafr	Pro-fibrotic response in the heart	Liang et al., 2018
	I/R	APF, upregulated	miR-188-3p	Competitive RNA	ATG7	Increased autophagy and myocardial injury	Wang et al., 2015b
		NRF, upregulated	miR-873	miRNA sponge	RIPK1/RIPK3	Enhanced myocardial necrosis	Wang et al., 2016
		RMRP, upregulated	miR-206	miRNA sponge	ATG3	Increased apoptosis and myocardial injury	Kong et al., 2019
**Metabolic heart disease**	Diabetic cardiomyopathy	H19 upregulated	miR-455	Competitive RNA	CTGF	Pro-fibrotic response in the heart	Huang et al., 2017

*Correlation, not confirmed as a direct miRNA target. APF, autophagy-promoting factor; ANP, atrial natriuretic peptide; BNP, brain natriuretic peptide; CaMKIIδ, Ca(2+)/calmodulin-dependent protein kinase II delta; CARL, cardiac apoptosis-related LncRNA; CHAR, cardiac hypertrophy-associated regulator; CHRF, cardiac hypertrophy related factor; CTGF, connective tissue growth factor; CYTOR, cytoskeleton regulator RNA; FADD, Fas-associated death domain; IKBKE, inhibitor of nuclear factor kappa-B kinase epsilon; Let-7d, Lethal 7 d; MIAT, myocardial infarction associated transcript; Mfn2, mitofusin 2; Myd88, myeloid differentiation primary response gene 88; NRF, necrosis-related factor; PFL, pro-fibrotic lncRNA PHB2: prohibitin 2; Ptafr, platelet-activating factor receptor; PTEN, phosphatase and tensin homolog; RIPK, receptor interacting protein kinase; TLR4, toll-like receptor 4; TAC, transverse aortic constriction; MI, myocardial infarction; I/R, ischemia/reperfusion; ISO, isoproterenol; PE, phenylephrine.

### LncRNA–miRNA Interactions in Cardiac Hypertrophy

With the recent finding that lncRNAs can modulate miRNA function, this appears as a novel target for their regulation in the heart. Furthermore, these lncRNA–miRNA interactions have been observed in different *in vivo* and *in vitro* models of cardiac hypertrophy. In this context, as previously mentioned, the lncRNA cardiac hypertrophy-related factor (CHRF) is upregulated *in vivo* in response to angiotensin II (Ang II) treatment and it is increased in human tissue from patients with heart failure (Wang et al., 2014b). The role of CHRF in cardiac hypertrophy is through its ability to act as a miRNA sponge to supress miR-489 activity. In the heart, the expression of miR-489 is profoundly reduced in response to Ang II and this results in the upregulation of its target, hypertrophic gene myeloid differentiation primary response gene 88 (Myd88), which has a pro-hypertrophic effect in the heart (Singh et al., 2012; Masè et al., 2019). Consistent with this, miR-489 forced overexpression *in vivo* shows reduced hypertrophic response under angiotensin II (Wang et al., 2014a). Additionally, CHRF is also upregulated in mice subjected to ISO injection. Interestingly, the increase of CHRF under hypertrophic stimulus correlates with an upregulation of miR-93, and ablation of CHRF in cardiomyocytes is sufficient to attenuate the ISO-induced hypertrophic responses via restoration of miR-93 expression. Furthermore, the kinase Akt3 was identified as the direct target for miR-93 in this model. Hence, the pro-hypertrophic activity of CHRF could be due to its sponging of miR-93, resulting in the upregulation of Akt3 activity and blunting miR-489 expression and activity, thus promoting the expression of hypertrophic genes such as Myd88 in response to hypertrophic stimuli (Wo et al., 2018).

Similarly, the lncRNA Plscr4 is also found to be upregulated in hypertrophic mice hearts (Lv et al., 2018). However, overexpression of Plscr4, in this case, leads to attenuation of cardiac hypertrophy, which appears to occur through altering the miR-214-Mitofusin 2 (Mfn2) pathway and maintaining mitochondrial homeostasis in mice subjected to pressure overload via Transverse Aortic Constriction (TAC) surgery. This was also shown in cardiomyocytes treated with angiotensin II. In response to pathological stimuli, upregulation of Plscr4 exerts an anti-hypertrophy effect by sponging miR-214, resulting in the attenuation of the inhibition of Mfn2 by miR-214 (Lv et al., 2018). Alterations in mitochondrial dynamics have been associated with pathological cardiac hypertrophy (Wüst et al., 2016) and recent reports have revealed that Mfn2 has a crucial role in the regulation of cardiac hypertrophy by regulating these processes under stress (Guan et al., 2016).

A recent study also identified the lncRNA cardiac hypertrophy-associated regulator (CHAR) as regulator of cardiac disease by acting as a competitive RNA *in vivo* (Zhang M. et al., 2019). CHAR expression is significantly decreased in hypertrophic conditions such as mouse models of pressure overload and *in vitro* angiotensin II-induced cardiomyocyte hypertrophy. Downregulation of CHAR by shRNA is sufficient to induce hypertrophic phenotypes, and this downregulation greatly exacerbated cardiac dysfunction induced by TAC. Conversely, forced overexpression of CHAR prevented hypertrophy. Mechanistically, CHAR acts by targeting miR-20b, a pro-hypertrophic factor which acts through inhibiting phosphatase and tensin homolog (PTEN), a known negative regulator of cardiac hypertrophy. PTEN downregulation AKT leading to hypertrophic responses to pathological stimulation (Zhang M. et al., 2019). In a similar fashion, the long non-coding RNA cytoskeleton regulator RNA (CYTOR) has also been described as a novel anti-hypertrophic modulator in the heart. However, unlike CHAR, CYTOR expression is markedly upregulated in response to pressure overload induced cardiac hypertrophy. CYTOR expression in the heart appears to be positively correlated with IKBKE expression, which has been reported to protect the heart from developing pathological cardiac hypertrophy through the IKKi and NF-κB signaling pathway (Yuan et al., 2019). Mechanistically, CYTOR functions as a ‘sponge’ for miR-155 to release IKBKE from miR-155-induced downregulation.

Several other lncRNAs have also been shown to regulate cardiac hypertrophy by functioning as miRNA sponges. Although most of the literature available on these corresponds to *in vitro* studies, the mechanistic insights provided by these will contribute to the understanding of cardiac pathology at the cellular level. The lncRNA myocardial infarction-associated transcript (MIAT) appears to be upregulated and act as a pro-hypertrophic factor after AngII and ISO treatment in cardiomyocytes. In response to AngII, MIAT acts as a molecular sponge of miR-93 which participates in the inactivation of the PI3K/Akt/mTOR pathway via targeting TLR4 in AngII-induced cardiac hypertrophy. Consistent with its hypertrophic effects, silencing MIAT results in a decreased expression of atrial natriuretic peptide and brain natriuretic peptide in ISO-treated NRVM cardiomyocytes. Under ISO treatment, MIAT acts by increasing P3000 expression levels via downregulation of miR-150p, resulting in cellular hypertrophy (Li et al., 2018c). MIAT has also been shown to promote the progression of AngII and isoproterenol induced cell hypertrophy *in vitro* by targeting miR-150 (Zhu et al., 2016). Similarly, ROR acts as a pro-hypertrophic factor via attenuation of miR-133 expression which results in augmented cardiac cell growth and ANP and BNP increased expression (Jiang et al., 2016), and H19 protects cardiomyocytes from phenylephrine-induced hypertrophy by targeting CaMKIIδ via miR-675 upregulation as miR-675 is encoded by H19 (Liu et al., 2016). These findings highlight the versatility of the functions of lncRNAs and miRNA-controlled cellular events. Thus, understanding these lncRNA–miRNA interactions and the modulation of different non-coding RNAs will help in the development of new cardiac hypertrophy treatments.

### LncRNAs–miRNA Interactions in Ischaemic Heart Disease

Acute myocardial infarction (MI) is caused by coronary artery occlusion. Insufficient blood supply results in the massive loss of cardiomyocytes by apoptotic and necrotic cell death (Krijnen et al., 2002) which results in impaired cardiac contractility due to pathological remodeling, such as fibrosis. Additionally, following MI, acute ischemia/reperfusion (I/R) injury leads to further cardiomyocyte death, triggered by reactive oxygen species (ROS) and metabolic dysfunction, leading to impaired cardiac function (Kalogeris et al., 2012). Several studies have highlighted the regulatory involvement of non-coding RNAs such as miRNAs, and more recently lncRNAs, as well as their interactions in the setting of myocardial infarction and reperfusion induced cardiac injury (Choi et al., 2014; Andreassi, 2018). The following sections review how these interactions are involved in MI-related events.

Among the pathological changes after MI, cardiac fibrosis is a characteristic and inevitable event. Cardiac fibrosis, although initially beneficial for heart function, results ultimately in impaired heart function (Kong et al., 2014). Generation and maintenance of the scar is key for the prevention of heart dilation at the infarct region. However, extracellular matrix deposition in the heart might lead to excessive collagen accumulation and ventricular wall stiffness which will greatly contribute to the development of heart failure (Frangogiannis, 2017). Fibrosis is finely regulated by various signaling pathways.

Different SNPs within the coding region for the lncRNA myocardial infarction associated transcript (MIAT) have been associated with increased MI risk (Ishii et al., 2006). *In vivo* studies showed that MI promotes the up-regulation of MIAT, which promotes fibrosis by reducing the availability of functional miR-24 in the heart through acting as a molecular sponge for miR-24 in cardiac fibroblasts (Qu et al., 2017). Further investigation showed that this pro-fibrotic effect is due to an increased expression of Furin, an activating factor of TGF-β1 that promotes cardiac fibrosis. Furin shows an reverse expression pattern compared to miR-24 and was identified as a miR-24 target. Consistently, *in vivo* models of MIAT knockdown show reduced infarct size and fibrosis and exhibit improved cardiac function in infarcted hearts. MIAT has also been shown to target several other anti-fibrotic miRNAs, including miR-29, miR-30, and miR-133 (Hobuß et al., 2019) further supporting its role in the promotion of cardiac fibrosis and remodeling.

Additionally, as described previously, MIAT expression is upregulated in cardiomyocytes under AngII treatment, participating as an inducer of cardiac hypertrophy by activation of the PI3K/Akt/mTOR pathway via TLR4 upregulation by sponging miR-93 in cardiomyocytes (Li et al., 2018b). Overall, MIAT-regulated fibrosis might be a promising therapeutic target for the alleviation of cardiac injury. Similarly, the lncRNA MALAT1 is upregulated in murine hearts in response to MI and in cardiac fibroblasts treated with angiotensin II. MALAT1, like MIAT1, shows a pro-fibrotic effect in the heart, and its knockdown via lentivirus-delivered MALAT1 siRNA is able to ameliorate the observed MI-impaired cardiac function, infarct size and ECM deposition *in vivo*. MALAT1 ablation also prevents AngII-induced fibrogenesis by halting fibroblast proliferation, collagen production, and α-SMA expression in neonatal cardiac fibroblasts. Mechanistically, MALAT1 acts as molecular sponge of miR-145. Similar to the MIAT-miR-24 axis, miR-145 inhibits the activation of TGF-β1 by decreasing the expression of Furin. Thus, MALAT1 promotes cardiac fibrosis by activating TGF-β1 dependent on miR 145 (Huang et al., 2019).

A recent study identified the lncRNA NONMMUT022555, or Pro-fibrotic lncRNA (PFL), as a novel fibrosis regulator. The expression of PFL is significantly higher in cardiac fibroblasts compared to cardiomyocytes and it is upregulated in mice subjected to MI promoting cardiac fibrogenesis mediated by fibroblasts. Mechanistically, PFL binds to the anti-fibrotic miRNA Let-7d acting as a sponge. Inhibition of Let-7d results in fibrogenesis via release of platelet-activating factor receptor (Ptafr) repression which leads to an increase in the expression of collagen1, α-SMA, FN1, and CTGF. Importantly, knockdown of PFL or forced expression of let-7d in MI mice markedly decreased the expression of profibrotic proteins and results in a decrease in collagen deposition and improved heart function in mice subjected to MI (Liang et al., 2018). Similarly, the expression of the lncRNA n379519 is significantly upregulated in the hearts of mice subjected to MI surgery and in TGF-b1-induced cardiac fibroblasts. Suppression of n379519 improves heart function and attenuates fibrosis in the myocardium. n379519 acts as a profibrotic lncRNA acting as a miR-30 sponge, which results in an upregulation of collagen I and collagen III expression. Interestingly, n379519 expression is also decreased when miR-30 is inhibited, suggesting a mutual regulation between the miR-30 and n379519 (Wang et al., 2018c).

Furthermore, in a model of diabetic cardiomyopathy, the antifibrotic miRNA, miR-455 was found to be significantly downregulated in both diabetic mice and in cardiac fibroblasts treated with AngII. *In vitro* studies revealed that miR-455 acts as an antifibrotic factor by targeting CTGF which results in a downregulation of proteins participating in fibrosis in cardiac fibroblasts. In primary mouse cardiac fibroblasts, the accumulation of collagen I and III observed after AngII treatment can be prevented by inhibiting miR-455 activity. The lncRNA H19 appears to have a regulatory role in this pathway as its expression is inversely correlated to that of miR-455. MiR-455 downregulation and expression of fibrosis associated proteins after AngII treatment can be recovered by ablation of H19 expression via siRNA in AngII-induced CFs. Thus, H19 could potentially modulate myocardial extracellular matrix accumulation through negative regulation of miR-455 by targeting CTGF (Huang et al., 2017).

During myocardial infarction, oxygen deficiency induces substantial cardiomyocyte loss by triggering apoptosis and necrosis (Krijnen et al., 2002). Several studies have highlighted the regulatory roles of non-coding RNAs in apoptosis in cancer and growing evidence have placed lncRNAs as crucial novel regulator of apoptosis and necroptosis in the infarcted heart. The role of lncRNAs such as MALAT1, UCA1, NRF, and H19 in survival has been discussed elsewhere (Zhou H. et al., 2019). Here we discuss the role of the lnRNA–miRNA axis in the regulation of cell death during MI, I/R and the failing heart.

The lncRNA Cardiac Apoptosis-Related LncRNA (CARL) regulates apoptosis by decreasing the expressions of Bax and the activity of Caspase-3, and by increasing Bcl-2 expression (Li et al., 2018d). CARL plays a critical role in cardiac apoptosis and mitochondrial dynamics regulating cardiac dysfunction via modulation of PHB2 expression in response to anoxia. PHB2 is important for maintaining the homeostasis of mitochondrial dynamics, and its expression is reduced during anoxia and ischemia-reperfusion in mitochondria (Wang et al., 2014b). PHB2 function is negatively regulated by miR-539. Furthermore, CARL was found to bind to miR-539 *in vivo*, acting as a functional sponge for miR-539, thus participating in maintaining the integrity of mitochondrial network dynamics via the CARL/miR-539/PHB2 axis. Consistent with this, adenoviral overexpression of CARL and the consequent downregulation of miR-539 results in increased PHB2 levels, which in turn is able to inhibit mitochondrial fission and apoptosis leading to smaller infarct size upon I/R *in vivo* (Wang et al., 2014b). Similarly, HOX antisense intergenic RNA (HOTAIR), downregulated by cardiac hypoxia or ischemia, was reported as a cardioprotective lncRNA (Gao et al., 2017). HOTAIR overexpression inhibits hypoxia/ischemia-induced apoptosis. Conversely, HOTAIR ablation results in a significant increase in cell death. Mechanistically, the cardioprotective role of HOTAIR might be explained by its interaction with miR-125 and miR-1, inhibiting their pro-apoptotic effects (Li et al., 2018a). In contrast, the lncRNA XIST was found to be upregulated in cardiomyocytes after infarction, protecting them from cell death by targeting miR-130a-3p, which has been linked to increased apoptosis and reduced proliferation (Zhou T. et al., 2019).

In addition to apoptosis, the induction of necrosis is prominent in the ischaemic heart (McCully et al., 2004). Recent studies have shown the importance of non-coding RNAs in necrosis. Fas-associated protein with death domain (FADD) triggers apoptosis through binding to death domain of members of the TNF family death receptors, including Fas and TNFR1 (Nagata, 1997). Additionally, FADD was shown to prevent formation of the RIPK1-RIPK3 complex, resulting in the inhibition of necrosis. The microRNA miR-103/107 participates in the downregulation of FADD-mediated necrosis in cardiomyocytes under oxidative stress, and in I/R injury in animal models (Wang et al., 2015a). In response to ischemic conditions, the lncRNA H19 inhibits myocardial necrosis by downregulating miR-103/107, relieving FADD downregulation (Wang et al., 2015a). In a similar fashion, the lncRNA necrosis-related factor (NRF) facilitates the programmed necrosis of myocardial cells under I/R condition by acting as a sponge to miR-873, and by doing so promotes RIPK1/RIPK3-dependent necrosis. Conversely, NRF ablation shows cardioprotective effects by preventing necrosis (Wang et al., 2016).

Abnormalities in the regulation of autophagy has also been linked to the pathogenesis of cardiovascular disease (Levine and Kroemer, 2008). The autophagy promoting gene ATG7 has also been reported to promote I/R-induced myocardial injury. *In vivo* and *in vitro* research has demonstrated that ATG7 is targeted by miR-188-3p, which results in the inhibition of autophagy and apoptosis. Under ischemia/reperfusion conditions, increased levels of the long non-coding RNA, autophagy-promoting factor (APF) regulates ATG7 expression by directly binding to miR-188-3p and inhibiting its activity. Furthermore, APF regulates autophagy through targeting miR-188-3p, which results in increased injury following MI *in vivo* (Wang et al., 2015b). Similarly, the lncRNA AK139328 modulates autophagy and apoptosis via inhibition of miR-204-3p during myocardial ischaemia/reperfusion injury in diabetic mice. Knockdown of lncRNA AK139328 relieved hypoxia/reoxygenation injury and protects against cardiac dysfunction in mice via inhibiting cardiomyocyte autophagy (Yu et al., 2018). In rats subjected to I/R, the upregulation of lncRNA RMRP is shown to aggravate myocardial injury and suppression of RMPR improves cardiac function and reduces levels of apoptosis. RMRP upregulates ATG3 expression and results in the activation of PI3K/AKT/mTOR pathway. Mechanistically, lncRNA RMPR acts by sponging miR-206, which targets ATG3, resulting in the subsequent ATG3 upregulation (Kong et al., 2019).

## miRNAs and lncRNAs as Potential Therapeutic Targets in CVD

As described above, cardiovascular disease still represents the number one cause of death worldwide (World Health Organization). With the recent advances in the understanding of the role of non-coding RNAs in physiology and pathology, and based on the growing evidence of the participation of non-coding RNAs in the pathogenesis of heart disease, their potential as novel therapeutic targets has become a matter of great interest not only for basic research, but also for pharmaceutical companies (Di Mauro et al., 2018). Cardiac remodeling is a progressive process accompanied by abnormal cell metabolism and function, remodeling of matrix components, and ultimately results in organ and systemic dysfunction (Schirone et al., 2017). As described above, several *in vitro* and *in vivo* studies have confirmed the critical roles of non-coding RNAs in cardiac remodeling. Cardiac-specific gene modulation experiments have shown a great potential to prevent and attenuate cardiac dysfunction or pathological progression in heart disease, thus highlighting the potential of non-coding RNAs as therapeutic targets. Here we will discuss the strategies currently available for non-coding RNA modulation and the potential issues that exist regarding the effectiveness of non-coding RNA-based therapies.

Drug and cell-based therapies in cardiac hypertrophy and ischemic injury have been found as effective intervention strategies (Machaj et al., 2019). However, the exact mechanisms and pathways involved are not always clear. Interestingly, some pharmacological agents have been described to act by regulating non-coding RNA expression and activities. An example of this is Losartan, an angiotensin II receptor antagonist used to treat hypertension. Losartan appears to diminish the effects of Ang II-induced fibrosis by restoring the lncRNA-NR024118 and Cdkn1c expression in cardiac fibroblasts (Jiang et al., 2015). It has also been shown that Atorvastatin, a hydroxymethylglutaryl coenzyme A reductase inhibitor used to treat dyslipidemia, possesses beneficial effects in patients with ischemic and non-ischemic induced heart failure. In a hypoxic cardiac progenitor cell (CPC) model, Atorvastatin treatment can protect progenitor cells from hypoxia-induced injury by inhibiting the expression of the lncRNA MEG3. MEG3 plays a role in CPC viability and proliferation by regulating HMGB1 expression through the inhibition of miR-22 (Su et al., 2018). These studies highlight the relevance of non-coding RNAs in the molecular mechanism of drugs currently used in the treatment of cardiovascular disease by protecting cardiac cells. Additionally, the direct modulation of the activity of non-coding RNA might present novel opportunities to develop more targeted and fine-tuned therapeutic approaches. Manipulation of the expression of miRNAs or lncRNAs with gene-based technologies has been effectively performed in animals, confirming the feasibility to ameliorate cardiac remodeling, myocardial inflammation and cell death, and heart hypertrophy using these genetic tools (Wang et al., 2018b).

It is possible to modulate the expression of non-coding RNAs in settings of disease progression in a tissue and cell-type specific fashion, which is promising for precision therapy. However, the challenge to transform the existing knowledge and methodologies used in research into therapies of relevance for clinical trials still persists. As non-coding RNAs are, compared to proteins, new potential therapeutic targets, the information about the effects of non-coding RNA based therapies is also limited and no extensive clinical data is available. Additionally, a key issue that needs to be addressed is that, particularly lncRNAs, have poor sequence conservation, presenting a clear challenge to extrapolate the findings observed in *in vivo* models to clinical based strategies requiring further optimization to ensure target selectivity and overall long term safety of non-coding RNA-based approaches (Poller and Fechner, 2010).

Another issue is that non-coding RNAs usually are degraded quickly and, as with other types of RNA, are damage prone (Amaral et al., 2013). Thus, exogenous gene delivery vectors, such as viruses are required for *in vivo* experiments (Di Mauro et al., 2018). Moreover, in the translation into clinical interventions, additional factors such as determination of bioavailability, optimization of delivery and distribution, safety, efficiency, concentration, stability, timing, etc. should be taken into consideration.

## Non-Coding RNA Modulation Strategies

In general, silencing of RNA is achieved by inhibitors, such as sequence-specific RNA interference (RNAi), antisense oligonucleotides (ASO) or the use of small molecule inhibitors. On the other hand, the overexpression or upregulation of non-coding RNA activities can be achieved by synthetic non-coding RNA mimics and other gene delivery strategies such as adeno-associated virus (Poller et al., 2010).

The use of antisense molecules has already been used in clinical trials targeting protein-coding mRNAs. An example, approved by the Food and Drug Administration, is Eteplirsen, a drug used to treat Duchenne muscular dystrophy caused by loss-of-function mutations in the *DMD* gene coding for dystrophin. Eteplirsen restores the translational reading frame of *DMD* through specific skipping of exon 51 in defective gene variants (Lim et al., 2017). In contrast to mRNAs and miRNAs, lncRNAs functions depend on their subcellular localization (Lennox and Behlke, 2016). Thus, for targeting lncRNAs this must be taken into consideration. For example, RNAi strategies will target RNA molecules in the cytoplasm, thus this approach might not be useful targeting nuclear lncRNAs. Additionally, tissue and cell type dependent expression has to be considered for targeted lncRNA modulation using specific delivery of antisense based therapies in different cardiovascular diseases.

### Non-coding RNAs Down-Regulation Strategies

miRNA target inhibitors and small molecule inhibitors: The binding of an inhibitor or protector interferes with the interaction of an miRNA with its target mRNA and therefore inhibits its silencing (Choi et al., 2007). Additionally, miRNA inhibition can be achieved with the use of low molecular weight compounds, known as small molecule inhibitors, that impair miRNA function by interfering with the miRNA maturation, processing and degradation machinery. An example of the use of these molecules in miRNA down-regulation is the use of diazobenzene 1 to inhibit the action of miR-21 (Gumireddy et al., 2008).

lncRNA small molecule inhibitors: As with miRNAs, use of small molecule inhibitors as an alternative strategy has to be taken into consideration for lncRNA modulation. Small compounds can inhibit the lncRNA activity by interfering with its binding to their targets (Colley and Leedman, 2009). The use of small molecules to modulate miRNA and lncRNA is currently being studied in cancer with promising results in the regulation of lncRNAs such as *HOTAIR* and MALAT1 (Arun et al., 2018). Interestingly, both these lncRNAs have been described to be implicated in cardiomyocyte survival and cardiac pathology, thus highlighting the potential use of this approach to treat lncRNA dysregulation in heart disease.

miRNA sponges and competitors: Similar to lncRNAs, artificial miRNA sponges can be generated using vectors with specific miRNAs binding sites. This will prevent the target miRNA activity by either sponging and decreasing the miRNA availability, competing for its binding target genes, or acting by interfering the miRNA–mRNA (or lncRNA) binding (Ebert et al., 2007).

#### Anti-miRNA Oligodeoxyribonucleotides (ASOs)

This strategy relies on small antisense oligonucleotides that act as miRNAs inhibitors by annealing to their guide strand. This results in either conformational changes that alter their function, or the degradation of the target miRNA (Broderick and Zamore, 2011). This approach has been extensively used in research and ASOs have been modified in order to improve their uptake, stability, and binding specificity. Addition of a 2′-*O*-methyl or 2′-*O*-methoxyethyl group to the RNA 2′-ribose results in the prevention of RISC nuclease degradation (Krützfeldt et al., 2005). Another important chemical modification, locked-nucleotide (LNA), enables an increased affinity and target specificity toward complementary single-stranded RNA molecules, by the formation of a methylene bridge between the 2′ oxygen with the 4′ carbon of the ribose ring (Thayer et al., 2019). However, the use of LNA oligonucleotides has been linked with liver damage (Burdick et al., 2014). This highlights the need for chemical refinement of ASOs before their use in the clinic.

#### LncRNA RNA-Interference (RNAi)

Although, RNAi-based technology is widely used for lncRNA downregulation, as mentioned above, it has to be taken into consideration that RNAi will mainly act in the cytoplasm. Thus, this approach will not be effective targeting lncRNAs that are active in the nucleus. The use of GapmeRs are a way to overcome this issue since they are able to block lncRNA activity in the nucleus via the nuclear endonuclease RNase H–dependent degradation (Lennox and Behlke, 2016). Structurally, GapmeRs consist of a DNA core flanked by two LNA sequences, which are complementary to the target RNA sequence. The chemical “lock” in the ribose backbone provides LNA with higher stability and increased endonuclease activity, resulting in greater knockdown efficiency (Swayze et al., 2006).

GapmeR-mediated silencing has been used to downregulate the lncRNA Chast, which mediates cardiac hypertrophy *in vivo* in mice and human cells *in vitro* (Viereck et al., 2016). The use of a GapmeR complimentary to Chast prevented and ameliorated pressure overload induced cardiac remodeling with no toxicological side effects. Similarly, *in vivo*, GapmeR-induced knockdown of the conserved lncRNA Wisp2 super-enhancer–associated RNA (Wisper) in mice subjected to MI results in reduced fibrosis and overall infarct size, and preserved cardiac function, further highlighting the potential use of lncRNA manipulation via GapmeRs for translation into clinics (Micheletti et al., 2017). Currently, the main limitations of these technologies are the formation of secondary structures that impair RNAi and GapmeR activity.

#### Virus-Mediated Gene Silencing

The adeno-associated virus (AAV) system has been used as a silencing strategy by carrying shRNA designed to specifically target lncRNAs in cardiac cells. An example of this is Meg3, a lncRNA which is highly conserved and enriched in both mouse and human hearts; increased expression of the lncRNA *Meg3* leads to profound cardiomyocyte apoptosis (Wu et al., 2018). *Meg3* inactivation by injection of an AAV9 system carrying *Meg3* shRNA into mouse hearts after MI preserved significantly cardiac function, suggesting that knockdown in a cardiomyocytes-specific manner can be achieved using AAV9 systems presenting a promising tool to treat cardiac disease in preclinical models.

### Non-coding RNAs Upregulation Strategies

In addition to inhibition or down-regulation of harmful non-coding RNAs, restoration of miRNA and lncRNA expression and activity has become a potential strategy for treatment in cardiac disease. The use of viral-mediated gene delivery, nanoparticles, or RNA mimics can be used to restore their expression when it is decreased in pathological contexts (Hobuß et al., 2019).

#### miRNA Mimics

miRNA mimics are synthetic small RNA molecules designed to be identical to a specific miRNA, thus restoring the expression of a miRNA that might be downregulated in a given pathological setting. This approach is the most widely used strategy for miRNA activity restoration. Similar to ASOs, mimics can also be modified to improve their activity, safety, delivery, and stability (Rupaimoole and Slack, 2017).

Virus-mediated gene expression: As a novel alternative, there is growing interest in the use of miRNA-expressing viral vectors (Xie et al., 2015). Lentivirus and adeno-associated virus (AAV) allow efficient gene delivery in a tissue specific manner. However, this approach still presents safety risks due to potential random viral DNA insertions into the genome or dysregulated overexpression of the delivered miRNA (Williams, 2007). Similar to the limitations in miRNA up-regulation strategies, lncRNA over-expression technologies are not fully developed. For example, the AAV vectors used for gene therapy in animal models have low packaging capacity and are not suited for lncRNA longer than 4 kb. Thus, more efforts are needed to develop novel strategies for lncRNA upregulation and restoration *in vivo.* Nevertheless, some studies have provided proof of principle for cardioprotective non-coding RNA upregulation via virus gene delivery in a preventative therapeutic approach in MI (Wang et al., 2014b).

Adeno-associated viruses (AAV) provide long-term, and efficient gene delivery into the heart. Furthermore, AAV systems have been used in clinical trials, such as the Calcium upregulation by percutaneous administration of gene therapy in cardiac disease (CUPID) trial which aims to restore SERCA2a by enzyme replacement via gene therapy (Jessup et al., 2011). However, less promising results have been observed in larger clinical trials. It is possible that modulation of single targets is not the optimal approach for treatment of many multifactorial diseases such as heart disease. Adenoviral overexpression of the previously described lncRNA Carl in MI models inhibits mitochondrial fission and cardiomyocyte loss via inhibition of miR-539 resulting in smaller infarct sizes *in vivo* (Wang et al., 2014b). However, overexpression of lncRNAs using viral gene delivery is still challenging and needs to overcome different limitations including the efficiency of infection and regulation of the lncRNA expression, which may differ depending on the pathological context. Considerations, such as whether transient or stable overexpression of a particular gene is needed, the magnitude of the response needed, the timing of the expression and cell specificity, have to be taken into consideration for therapeutic treatment.

## Future Perspectives

Despite the evidence of the role of lncRNAs and miRNAs in the regulation of ER stress in the heart, to date the interaction between these regulatory RNAs in the context of cardiac disease remains unknown. Evidence from other disease models highlights the potential relevance of this new level of regulation in the promotion of the UPR in order to alleviate ER stress. We propose that the study of lncRNA–miRNA–mRNA axis and other forms of non-coding RNA interactions in the context of ER stress in cardiomyocytes could give insights into new levels of regulation of the progression of cardiac hypertrophy and ischemic damage.

For instance, the lncRNA MIAT, previously described in models of MI and hypertrophy, participates in the regulation of the ER chaperone, GRP78. Both MIAT and GRP78 levels are increased in Müller cells under high-glucose treatment (Xu et al., 2019). Mechanistically, MIAT acts as a molecular sponge for microRNA-379, which targets GRP78 mRNA for its degradation. Similarly, another study in a high glucose diabetic model shows an upregulation of around 40 miRNAs within the miR-379 megacluster. Interestingly, this megacluster is hosted by the lncRNA lnc-MGC which is upregulated by ER stress (Kato et al., 2016). The miRNAs in the cluster, as mentioned above, target different groups of genes participating in fibrosis, protein synthesis, and ER stress, resulting in hypertrophy via dysregulation of protein synthesis and extracellular matrix accumulation related to diabetic nephropathy. Thus, in pathological conditions, the increase in lncRNA MGC and the miR-379 cluster further enhance ER stress and nephropathy phenotypes by inhibiting the miR-379 cluster targets. Additionally, Xbp1 splicing and Atf3 downregulation occurs via upregulation of miR-494, one of the miR-379 cluster miRNAs, which participates in the upregulation of Chop in response to high glucose or TGF-β1 in mouse mesangial cells (Kato et al., 2016). Furthermore, it was observed that miR-379 cluster upregulation in diabetes contributes to ER stress via loss of Edem3. Therapeutically, as previously described, the use of GapmeRs is an attractive and effective strategy for the regulation of cellular functions mediated by LncRNAs. Inhibition of the miRNA cluster by targeting its host lnc-MGC using the GapmeR MGC10 was able to ameliorate matrix accumulation and glomerular hypertrophy in mice by restoring the expression of the cluster miRNA targets.

In a diabetic nephropathy model the ER stress response can be regulated by the lncRNA LINC01619 acting as a molecular sponge for miR-27a (Bai et al., 2018). miR-27a targets forkhead box protein O1 (FOXO1) for its degradation leading to ER stress activation and resulting in podocyte injury. Inhibition of miR-27a can effectively increase the expression level of FOXO1 and decrease the ER stress markers CHOP and GRP78 in podocytes under high glucose-triggered ER stress, further highlighting the role of lncRNA–miRNA–mRNA regulation in metabolic disease. Additionally, modulation of the ER stress response has also shown to be beneficial in a model of osteosarcoma. The transcription factor ZBTB7A appears to have a pro-survival role in cells undergoing ER stress via suppression of the lncRNA GAS5 by binding to its promoter region and transcriptionally suppressing its expression. Pharmacological induction of ER stress results in the downregulation of ZBTB7A accompanied by sustained apoptosis. Mechanistically, ER stress triggers the induction of miR-663a which targets the 3′UTR of ZBTB7A for its downregulation, resulting in an upregulation of GAS5 leading to ER stress-induced cell apoptosis (Zhang L. et al., 2019).

Overall, since ER stress plays an essential role in cardiac physiology and pathology, finding new approaches to modulate the ER stress responses in the heart is essential to develop novel therapeutic approaches. The further study of the modulation of key stress pathways via the genetic or pharmacological regulation of non-coding RNAs could provide the ability to fine tune these responses in order to enhance pro-survival mechanisms and to minimize the cell and tissue damage in the progression of disease.

## Conclusion

**FIGURE 2:**
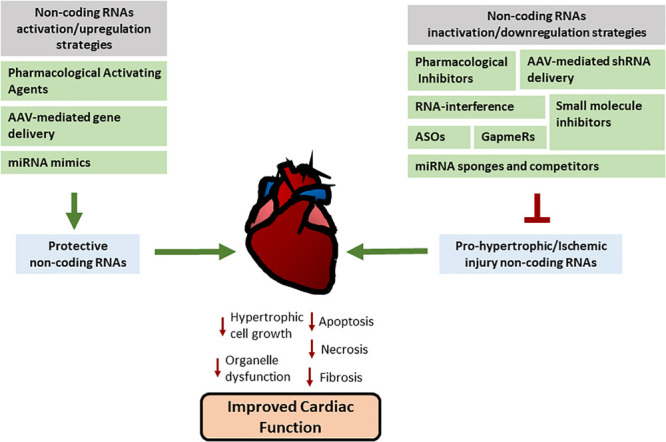
Therapeutic approaches used for non-coding RNA modulation. Different pharmacological and genetic approaches are being developed in order to regulate miRNAs and lncRNAs activities or manipulate their expression. Their main aim is to increase the activity or upregulate/restore the expression of non-coding RNAs with cardiac protective effects (on the left) or inhibit or downregulate the expression of non-coding RNAs that have a detrimental effect in heart pathology (on the right) in order to reduce the progression of pathological processes. See text for more details.

In summary, a great number of non-coding RNAs are dynamically regulated upon initiation and progression of cardiovascular disease. Many non-coding RNAs have important biological functions and several *in vivo* experiments have revealed that modulation of miRNAs and lncRNAs offers promising new therapeutic approaches (Figure 2). However, inhibition or activation of non-coding RNA function and its consequences still requires further investigation. Refinement on the current approaches and the development of novel strategies will allow in the future clinical translation for non-coding RNA-based therapy and its use in the treatment of different pathologies including cardiovascular disease.

## Author Contributions

LC and PB development of conceptual ideas and general outline and writing and edition of the manuscript. HC provided critical feedback and editing of the manuscript. XW development of conceptual ideas and provided critical feedback. All authors contributed to the article and approved the submitted version.

## Conflict of Interest

The authors declare that the research was conducted in the absence of any commercial or financial relationships that could be construed as a potential conflict of interest.
